# Lymphopenia in Esophageal Cancer: What Have We Learned?

**DOI:** 10.3389/fonc.2021.625963

**Published:** 2021-03-11

**Authors:** Jia-Lin Wang, Rong Ma, Wei Kong, Ren Zhao, Yan-Yang Wang

**Affiliations:** ^1^Department of Radiation Oncology, General Hospital of Ningxia Medical University, Yinchuan, China; ^2^Cancer Institute, Ningxia Medical University, Yinchuan, China; ^3^Graduate School, Ningxia Medical University, Yinchuan, China

**Keywords:** lymphopenia, esophageal cancer, predictor, prognosis, immunotherapy

## Abstract

Lymphopenia caused by disease or treatment is frequent in patients with cancer, which seriously affects the prognosis of these patients. Immune checkpoint inhibitors (ICIs) have garnered attention as one of the most promising strategies for the treatment of esophageal cancer (EC). The status of the immune system, such as, the lymphocyte count, is now considered to be an important biomarker for ICI treatments. Recognition of the significant impact of the lymphocyte count on the survival of patients with EC in the era of immunotherapy has revived interest in understanding the causes of lymphopenia and in developing strategies to predict, prevent and eliminate the adverse effect of lymphopenia. Here, we review what we have learned about lymphopenia in EC, including the prognostic and predictive value of lymphopenia in patients with EC, the predictors of lymphopenia, and the strategies to ameliorate the effect of lymphopenia in patients with EC.

## Introduction

**Figure 1:**
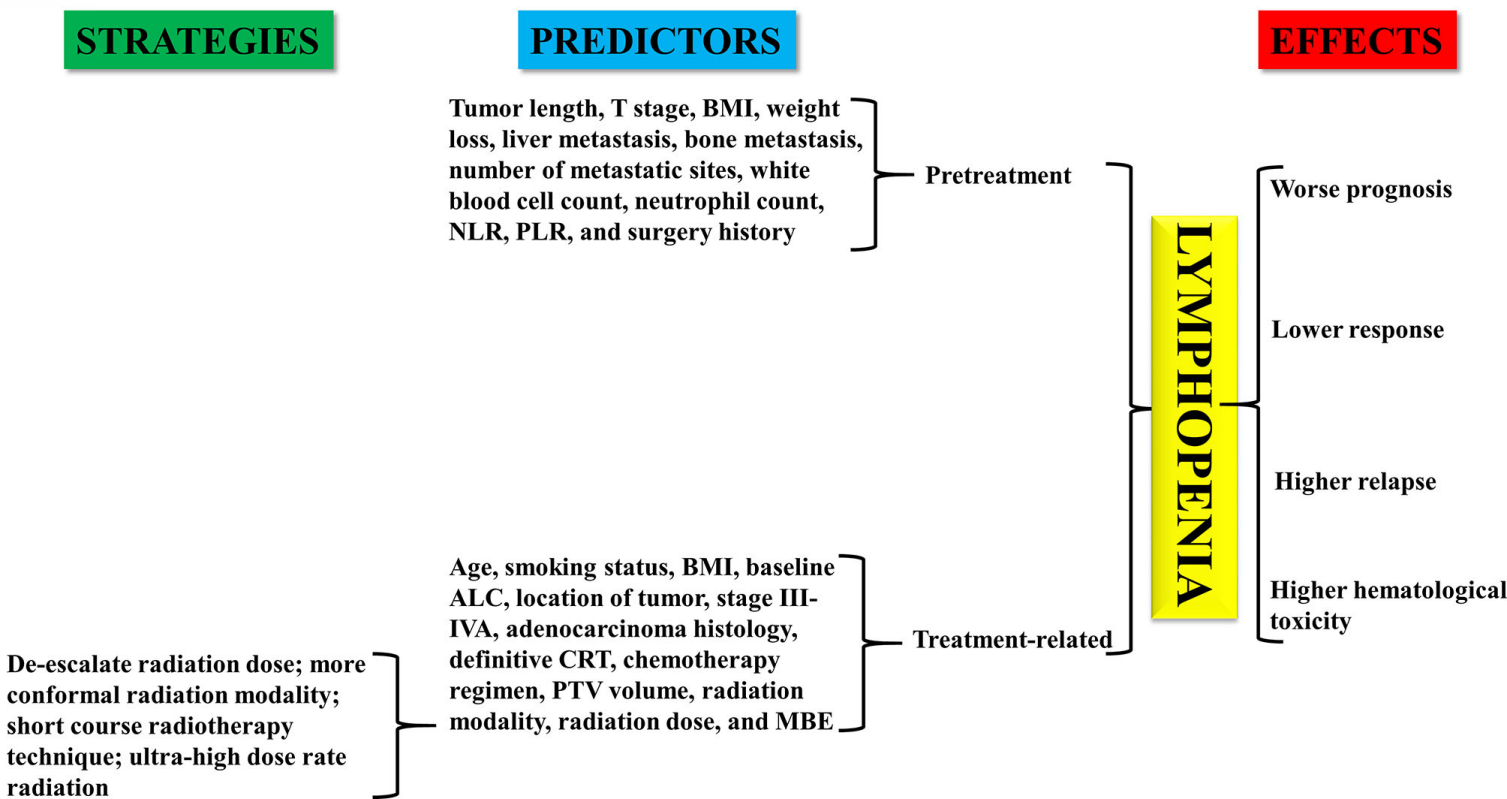
Effect of lymphopenia on patients with esophageal cancer. Predictors and coping strategies of lymphopenia in esophageal cancer.

The immune system plays a critical role in controlling and eradicating cancer (1–3). Peripheral blood lymphocytes are considered to be crucial components of the immune system and have the function of mediating cellular immunity against neoplastic cells (4–6). Previous studies have shown that baseline or treatment-induced lymphopenia is associated with the short-term survival of various cancers, including esophageal cancer (EC) (7, 8). Therefore, lymphopenia may be a useful marker for the management of EC. The progress of checkpoint-directed immunotherapy provides additional motivation for exploring the role of lymphopenia in the treatment of EC (9–11). In this minireview, we mainly summarize the prognostic and predictive value of lymphopenia in EC. The predictors of lymphopenia and the strategies to eliminate the effect of lymphopenia on the management of EC will also be discussed (**Figure 1**).

## The Prognostic Role of Lymphopenia in EC

Although the mechanisms responsible for the interaction between lymphopenia and the treatment outcome of EC remain largely unclear (12), increasing clinical data have shown that a low absolute value of lymphocytes is associated with poor prognosis of EC.

Surgery is the recommended treatment approach for patients with EC (13), especially those with early-stage cancer. A retrospective analysis was performed to evaluate the prognostic value of preoperative lymphopenia in patients with esophageal squamous cell carcinoma (ESCC) undergoing esophagectomy (14). The incidence of lymphopenia (lymphocyte count <1.0Giga/L) in this cohort was 16.6%. The cancer-specific survival (CSS) rate at 5 years was significantly lower in the patients with lymphopenia (21.6% vs. 43.8%, P = 0.004). Multivariate analysis demonstrated that lymphopenia was an important predictor for CSS. This study showed that lymphopenia was associated with prognosis in patients with EC undergoing surgery.

The prognostic value of lymphopenia in patients with EC treated by chemoradiotherapy (CRT) was also explored. A total of 504 patients with stage I-III EC treated with neoadjuvant or definitive CRT were retrospectively analyzed (15). The incidences of grade (G) 1, 2, 3, and 4 absolute lymphocyte count (ALC) nadirs during CRT were 2%, 12%, 59%, and 27%, respectively. G4 ALC nadir (G4 nadir) was significantly correlated with shorter overall and progression-free survival (PFS). The median overall survival (OS) of patients with G4 nadir and G0-2 nadir disease was 2.8 and 5.0 years (P = 0.027), respectively. The median PFS of patients with G4 nadir and those without was 1.1 and 5.1 years (P < 0.001), respectively. The results were confirmed in another study. A total of 189 patients with ESCC were included in the study (16). All patients received definitive radiotherapy combined or not with chemotherapy. ALC values were assessed before, during, and after radiotherapy. During the study period, 110 patients exhibited a low ALC nadir (≤ 0.38 × 10^3^ cells/µl). Compared with patients with a high ALC nadir, patients with a low ALC nadir had unfavorable OS [hazard ratio (HR), 2.08; P < 0.001], PFS (HR, 1.69; P = 0.0048), and local recurrence-free survival (LRFS) (HR, 1.81; P = 0.0053).

Moreover, lymphocyte count seems to have some effect in the treatment of patients with metastatic ESCC. Kou et al. (17) investigated the influence of pretreatment lymphopenia on the efficacy and toxicity of first-line chemotherapy in patients with metastatic ESCC. The results demonstrated that pretreatment lymphopenia was found in 19.1% of patients with metastatic ESCC. Kaplan–Meier curves showed that patients with ESCC with pretreatment lymphopenia had a significantly shorter OS than those without lymphopenia (8.2 vs. 12.7 months; P = 0.020).

The contribution of dynamic changes in lymphocyte counts to the prognosis of patients with ESCC was also assessed recently (18). This analysis was limited to patients with stage I–III EC who received CRT followed or not by surgery. A total of 38.9% of the enrolled patients had G4 lymphopenia during CRT. Multivariate analysis showed that G4 lymphopenia was an independent prognostic factor. The 5-year OS rates were 35.4% G4 vs. 51.8% G0-3 (P < 0.001); the 5-year PFS rates were 30.1% G4 vs. 40.7% G0-3 (P = 0.002); the 5-year LRFS rates were 31.9% G4 vs. 45.4% G0-3 (P = 0.001); and the 5-year distant metastasis-free survival (DMFS) rates were 34.2% G4 vs. 46.3% G0-3 (P < 0.001). After the first follow-up, 53.8% of the patients recovered (Gr0-1). However, the recovery of lymphocytes did not indicate a better prognosis. The 5-year OS rate of patients with G4 lymphopenia during CRT and recovery (Gr0-1) afterward was lower than that of the G0-3 unrecovered group (Gr2-4) (36.6% vs. 51.9%, P = 0.027). In addition, the degree of lymphopenia during CRT did not affect the recovery ability of lymphocytes after the treatment.

ICIs, especially programmed cell death protein-1 (PD-1) and PD ligand 1 (PD-L1), are an emerging therapy modality for EC (10, 11). Although there is no direct evidence of lymphopenia and prognosis in patients with EC treated with ICIs, some studies have evaluated the prognostic role of the neutrophil-to-lymphocyte ratio (NLR) in patients with EC treated with ICIs. A retrospective study (3) showed that a high NLR was a statistically significant prognostic factor associated with poor PFS and OS in patients with recurrent or metastatic ESCC treated with PD-1/PD-L1 blockers. The median PFS of patients with low and high NLRs was 2.8 months and 1.4 months, respectively (P = 0.001). The median OS of patients with low and high NLRs was 10.4 months and 3.0 months, respectively (P < 0.001). In another study (19), the effect of the derived NLR [absolute neutrophil count/(white blood cell concentration - absolute neutrophil count)] on prognosis was evaluated in patients with noncolorectal gastrointestinal cancer who received ICIs. The OS of patients with high or low dNLR values was 4.2 months and 10.43 months, respectively (P < 0.001).

## The Predictive Role of Lymphopenia in EC

An increasing number of studies have shown that lymphopenia can predict the response and toxicity of treatment in patients with EC, so it can partly explain why lymphopenia is related to the treatment outcomes of these patients.

Neoadjuvant CRT is becoming the standard for the treatment of EC (20–22). Pathologic complete response (pCR) during CRT has been considered to be a favorable prognostic factor for patients with EC. The role of lymphopenia in predicting pCR was confirmed in a study of 313 patients with EC who received neoadjuvant CRT followed by surgery (23). A total of 27.8% of patients achieved pCR. High ALC was defined as a nadir of ≥0.35 × 10^3^/µl. Patients with a high ALC nadir had a higher pCR rate [odds ratio (OR) 1.82, P = 0.024]. In another study, Li et al. (24) retrospectively analyzed the correlation between treatment-related lymphopenia and pCR of neoadjuvant CRT in patients with ESCC. All enrolled patients received neoadjuvant CRT, followed by a radical esophagectomy. A total of 43.2% of the patients achieved pCR in histopathological examination. G4 lymphopenia was observed in 21.8% of patients. Patients with G4 lymphopenia had a significantly lower pCR rate than those without (G0–2 vs. G4, P = 0.001; and G3 vs. G4, P = 0.007). Additionally, the results also showed that patients with G4 lymphopenia experienced a higher relapse rate (45.8% vs. 28.5%, P = 0.023). These data suggest that treatment-related lymphopenia is an effective predictor of pathological response and disease recurrence in EC.

Recently, a study has also been conducted to assess the significance of lymphopenia in predicting response rates for patients with locally advanced EC who received radical CRT (25). Patients who were treated with definitive CRT for locally advanced ESCC were eligible for this study. During CRT, 31% of patients experienced treatment-related lymphopenia (total lymphocyte count <200 cells/mm^3^), and 21.7% of patients achieved clinical CR. The CR rate for patients with lymphopenia was lower than the CR rate for the remaining patients with higher lymphocyte counts (11.2% vs. 26.4%, P = 0.003). Multivariate analysis showed that treatment-related lymphopenia was the only independent factor correlated with a lower CR rate (P = 0.043). This study further confirmed the value of lymphopenia in predicting the response rate of patients with EC.

In addition to predicting the response rate, lymphopenia is also involved in the prediction of toxicity induced by chemotherapy in EC. A total of 215 patients with metastatic ESCC were included in this retrospective study (17), and 19.1% of patients exhibited pretreatment lymphopenia. The study showed that patients with lymphopenia were more likely to develop G3-4 hematological toxicity during chemotherapy (46.3% vs. 31.0%; P = 0.048). However, a correlation between lymphopenia and G3–4 nonhematological toxicity was not found.

Furthermore, the recovery of lymphopenia can also be used as a predictor for treatment relapse in patients with EC. A total of 198 patients with EC undergoing esophagectomy were included in the analysis (26). The results revealed that compared with those who recovered or never dropped, the recurrence rate was significantly higher in patients with EC with persistent lymphopenia (43% vs. 14%; P = 0.0017) (**Table 1**).

**Table 1:** Summary of prognostic or predictive role of lymphopenia in patients with esophageal cancer.

N	TNM stage	Lymphopenia metric	% with lymphopenia	Treatment	Endpoints (lymphopenia vs. no lymphopenia)	Reference
307	I-IVA	lymphocyte count <1.0Giga/L	16.6%	Surgery ± CT or RT	5yr cancer-specific survival 21.6% vs. 43.8% (P = 0.004)	(14)
504	I-III	lymphocyte count <200 cells/µl	26.6%	CRT	OS 2.8 yr vs. 5.0 yr (P = 0.027), PFS 1.1 yr vs. 5.1 yr (P < 0.001)	(15)
189	I-IVA	lymphocyte count≤ 0.38 × 10^3^/µl	58.2%	RT	OS (HR, 2.08; P < 0.001), PFS (HR, 1.69; P = 0.0048), LRFS (HR, 1.81; P = 0.0053)	(16)
215	IVB	lymphocyte count <1 × 10^9^/L	19.1%	CT + RT	OS 8.2 mo vs. 12.7 mo (P = 0.020), G3-4 hematological toxicity 46.3% vs. 31.0% (P = 0.048)	(17)
755	I-III	lymphocyte count <200/µl	38.9%	CRT ± Surgery	5 yr OS 35.4% vs. 51.8% (P < 0.001), PFS 30.1% vs. 40.7% (P = 0.002), LRFS 31.9% vs. 45.4% (P = 0.001), DMFS 34.2% vs. 46.3% (P < 0.001)	(18)
49	IVB	NLR > 6.40	50%	PD-1/PD-L1-blockage	PFS 1.4 mo vs. 2.8 mo (P = 0.001), OS 3.0 mo vs. 10.4 mo (P < 0.001)	(3)
160	IVB	dNLR ≥ 3	31.2%	PD-1, PD-L1 or CTLA-4 blockage	OS 4.2 mo vs. 10.43 mo (P < 0.001)	(19)
313	I–IVA	lymphocyte count < 0.35 × 10^3^/µl	IMRT 62%, PBT 44%	CRT + Surgery	pCR rate OR 1.82 (P = 0.024)	(23)
220	II-III	lymphocyte count <200/µl	21.8%	CRT + Surgery	pCR rate OR 3.134, (P = 0.003), relapse rate 45.8% vs. 28.5% (P = 0.023)	(24)
286	II–IVA	lymphocyte count <200/µl	31%	CRT	CR rate 11.2% vs. 26.4% (P = 0.003)	(25)
198	0-IV	lymphocyte count <1.0 × 10^9^/l	76.8%	Surgery	Recurrence rate 43% vs. 14% (P = 0.0017)	(26)

CR, complete response; CRT, chemoradiotherapy; CT, chemotherapy; CTLA-4, cytotoxic T lymphocyte antigen 4; DMFS, distant metastasis-free survival; dNLR, derived NLR; G, grade; HR, hazard ratio; IMRT, intensity-modulated radiation therapy; LRFS, local recurrence-free survival; N, number; NLR, neutrophil-to-lymphocyte ratio; OR, odds ratio; OS, overall survival; PBT, proton beam therapy; pCR, pathologic complete response; PD-1, programmed cell death protein-1; PD-L1, PD ligand 1; PFS, progression-free survival; RT, radiotherapy.

## Predictors of Lymphopenia in EC

Given the association between lymphopenia and worse survival or poor response as previously mentioned, identifying reliable predictors of lymphopenia can minimize the adverse effects and select high risk EC patients for risk mitigating interventions.

The predictors of pretreatment lymphopenia of EC include larger tumor length, late T stage, body mass index (BMI) ≤18.5 kg/m^2^, and weight loss ≥3 kg in the previous 3 months (25). Additionally, for stage IV EC, liver metastasis, bone metastasis, number of metastatic sites, white blood cell count, neutrophil count, NLR, platelet-lymphocyte ratio (PLR), and surgery history are significantly associated with the risk of pretreatment lymphopenia (17).

Compared with pretreatment lymphopenia, more studies have focused on the analysis of predictive factors of treatment-related lymphopenia in EC (16, 24, 27–29). Factors that predict lymphopenia during treatment in EC include advanced age, nonsmoking history, lower BMI, decrease in baseline ALC, distant location of tumor, stage III-IVA, adenocarcinoma histology, definitive CRT, chemotherapy regimen (paclitaxel+5-fluorouracil), larger planning target volume (PTV), radiation modality (photon-based vs. proton-based), higher radiation dose (≥40Gy), and increased mean body dose exposure.

Radiotherapy is an important component in the treatment of EC (30, 31). Among the predictive factors of treatment-induced lymphopenia, the factors related to radiotherapy have been extensively studied recently (32). These studies have shown that the degree of lymphopenia caused by radiotherapy depends on the dose/volume of blood flow as well as organs rich in lymphatics and lymphocytes in the radiation field (33). Due to the anatomic position of the esophagus near the heart and the contribution of the heart to lymphocyte circulation, the relationship between heart dose and the severity of lymphopenia in patients with EC during radiotherapy was explored. The results revealed that the percentage of heart volume exposure to 10 Gy (heart V10) and 20 Gy (heart V20) were predictors of radiation-induced lymphopenia in patients with EC (16). In addition to the heart, irradiated dose and volume of the lung and liver are also strongly associated with lymphocyte destruction. Xu et al. (34) found that lung V10 and heart V10 were significantly associated with G4 lymphopenia in patients with ESCC treated with definitive CRT. They suggested that minimizing low-dose areas in the lung and heart could reduce radiation-induced lymphopenia. Jin et al. (35) developed a model to calculate the effective dose to immune cells (EDIC) in the heart, lung and liver of patients with EC who were treated with concurrent CRT. The correlation between EDIC and lymphopenia was analyzed. Patients with higher EDIC values (> 4 Gy) were more likely to experience G4 lymphopenia during treatment (67.3% vs. 40.8%, P < 0.001).

The spleen is the largest secondary immune organ in the body. Saito et al. (36) showed that spleen V5, V10, V20, and V30 and the mean splenic dose were significant predictors of treatment-induced lymphopenia in EC. When the mean splenic dose increased by 1 Gy, the predicted ALC decreased by 2.9%.

Moreover, there is evidence that lymphopenia is closely correlated with thoracic vertebral bodies receiving radiation during CRT for EC. Anderson et al. (27) explored the effect of the thoracic vertebral dose on ALC in patients with EC treated with radiotherapy. They first defined TVS5-40 (thoracic vertebral volume spared 5–40 Gy), that is, thoracic vertebra volume (TV) minus TV5-TV40. There was a significant correlation between TVS5-40 and higher lymphocyte nadirs during treatment. Another study confirmed the predictive value of the vertebral dose in radiotherapy-induced lymphopenia for EC (28). The results showed that increasing the vertebral volume of ≥10Gy, ≥20Gy, ≥30Gy or the mean vertebral body dose was associated with G4 lymphopenia in patients with EC who received definitive or neoadjuvant CRT.

## Coping Strategies for Lymphopenia in EC

Owing to advances in the etiology of radiation-induced lymphopenia, strategies for dealing with this type of lymphopenia have been systematically studied (37). As mentioned earlier, factors affecting radiation-induced lymphopenia include the radiation dose, target volume, and fraction numbers. Therefore, one of the strategies to address radiation-induced lymphopenia is to de-escalate the radiation dose. It was shown that there was no change in the lymphocyte count following neoadjuvant chemotherapy, but a significant reduction was noted after the initiation of thoracic radiotherapy (38). It is therefore possible to apply other concurrent treatment modalities without causing lymphopenia with radiotherapy, which could limit the radiation dose, maintain the treatment intensity and minimize the negative effect of radiation on lymphocytes.

Radiotherapy modality and technique may have an impact on the severity of lymphopenia. The second strategy is to use a more conformal radiation modality (39). New planning/treatment strategies, such as proton therapy or spleen-sparing treatment plans, can reduce unintentional exposure to circulating blood pools and secondary lymphoid organs, which are contributors to radiation-induced lymphopenia. Proton therapy does not penetrate the whole body and may lead to less blood exposure, which is associated with less lymphopenia than photon therapy (40). Shiraishi et al. (41) found that proton beam therapy could reduce the incidence of G4 lymphopenia from 40.4% to 17.6% in patients with EC treated with neoadjuvant CRT compared with intensity-modulated radiotherapy (IMRT). This result was confirmed in a similar study (42). The incidence of G4 lymphopenia in patients with EC treated with photon therapy and proton therapy was 60% and 24%, respectively. In addition to proton therapy, the arrangement of radiation beams can also be improved to minimize doses to specific organs, such as the spleen, which has a large pool of lymphocytes, thereby reducing the risk of lymphopenia in patients with EC.

With the increase in fraction numbers, the proportion of irradiated circulating lymphocytes will be enlarged, thus increasing the risk of radiation-induced lymphopenia. The use of short-course radiotherapy techniques such as stereotactic body radiotherapy (SBRT), can minimize radiation exposure to normal tissue (43). In non-small cell lung cancer, clinical studies have shown that SBRT treatment leads to less lymphopenia (44). Therefore, the selection of SBRT treatment for patients with EC with indications is also one of the strategies to reduce the incidence of lymphopenia. In addition, increasing the radiation dose rate and shortening the delivery time can further reduce the killing effect of X-rays on circulating immune cells. For example, FLASH radiotherapy provides large doses of radiation in a very short time (<0.1 s), which can avoid the unintentional exposure to lymphocytes. The killing rate of circulating immune cells decreased from 90%–100% with the conventional dose rate to 5%–10% with the ultrahigh dose rate (45).

## Conclusion

Lymphopenia in patients with EC seriously affects their response to treatment, toxicity and survival, which emphasizes the importance of the immune status in improving cancer treatment outcomes. The discovery of predictors of lymphopenia, especially for radiation-induced lymphopenia, may open potential therapeutic strategies to prevent or mitigate lymphopenia. Several “immune-sparing” strategies have been used in radiotherapy for patients with EC. However, the development of more robust methods to counter lymphopenia in patients with EC depends on the understanding of the mechanisms of lymphopenia. Before the realization of the mechanisms of lymphopenia, several studies have shown that lymphopenia can also be used as a biomarker to identify which patients may benefit from checkpoint inhibitors or other lymphocyte-mediated immunotherapies (46). In addition, although there are many studies on the role of lymphopenia in EC, the different cutoff values of lymphopenia in various studies may affect the consistency of the conclusions. Seeking a consistent cutoff value of lymphopenia in patients with EC is also an urgent problem to be solved before it is widely used in clinical practice.

## Author Contributions

J-LW, RM, and Y-YW researched the data, wrote the review, and designed the figure. WK, RZ, and Y-YW reviewed and revised the manuscript. All authors contributed to the article and approved the submitted version.

## Funding

This work is supported by the National Natural Science Foundation of China (82060433).

## Conflict of Interest

The authors declare that the research was conducted in the absence of any commercial or financial relationships that could be construed as a potential conflict of interest.
